# Pax7 Lineage Contributions to the Mammalian Neural Crest

**DOI:** 10.1371/journal.pone.0041089

**Published:** 2012-07-27

**Authors:** Barbara Murdoch, Casey DelConte, Martín I. García-Castro

**Affiliations:** 1 Biology Department, Eastern Connecticut State University, Willimantic, Connecticut, United States of America; 2 Department of Molecular, Cellular and Developmental Biology, Yale University, New Haven, Connecticut, United States of America; Instituto de Medicina Molecular, Portugal

## Abstract

**Background:**

Neural crest cells are vertebrate-specific multipotent cells that contribute to a variety of tissues including the peripheral nervous system, melanocytes, and craniofacial bones and cartilage. Abnormal development of the neural crest is associated with several human maladies including cleft/lip palate, aggressive cancers such as melanoma and neuroblastoma, and rare syndromes, like Waardenburg syndrome, a complex disorder involving hearing loss and pigment defects. We previously identified the transcription factor Pax7 as an early marker, and required component for neural crest development in chick embryos. In mammals, Pax7 is also thought to play a role in neural crest development, yet the precise contribution of Pax7 progenitors to the neural crest lineage has not been determined.

**Methodology/Principal Findings:**

Here we use Cre/loxP technology in double transgenic mice to fate map the Pax7 lineage in neural crest derivates. We find that Pax7 descendants contribute to multiple tissues including the cranial, cardiac and trunk neural crest, which in the cranial cartilage form a distinct regional pattern. The Pax7 lineage, like the Pax3 lineage, is additionally detected in some non-neural crest tissues, including a subset of the epithelial cells in specific organs.

**Conclusions/Significance:**

These results demonstrate a previously unappreciated widespread distribution of Pax7 descendants within and beyond the neural crest. They shed light regarding the regionally distinct phenotypes observed in Pax3 and Pax7 mutants, and provide a unique perspective into the potential roles of Pax7 during disease and development.

## Introduction

Unique to vertebrates, multipotent neural crest cells are found during early embryonic development, and in limited but specific regions in the adult [1]–[4]. The neural crest arises between the neural plate and non-neural ectoderm, and migrates extensively to numerous locations throughout the body, where they contribute to a wide range of cell and tissue derivatives.

Neural crest cells can be operationally defined based on their position within the anterior/posterior neural axis; cells at a given axial level follow distinct migratory routes and differentiate into a predictable array of progeny at their final destination [5]–[7]. From anterior to posterior, the axis can be roughly divided into three groups: the cranial, cardiac (also termed vagal), and trunk (including the sacral) neural crest. The cranial crest migrates dorsolaterally into the head region to produce cells including neurons, glia, and craniofacial mesenchyme such as various connective tissues (muscle, bones and cartilage) [6], [8]–[10]. Also produced by the cranial neural crest are thymic cells, the tooth primordia, and the bones of the middle ear and jaw. A forward migration of the cranial crest can also deposit cells into the frontonasal process. The cardiac neural crest cells migrate to the 3^rd^, 4^th^ and 6^th^ pharyngeal arches, which contribute to the muscle and connective tissue of the walls of the aortic arch arteries and the aorticopulmonary septum [11]. In the trunk, neural crest cells migrate either dorsolaterally to produce melanocytes, or ventrolaterally to produce cells of the peripheral nervous system (the dorsal root, sympathetic and parasympathetic (enteric) ganglia) [5]. The sacral neural crest, found in mice posterior to somite 28, also contributes to cells of the peripheral ganglia [7], [12].

The broad spectrum of neural crest tissue derivatives is equally matched by their excessive participation in disease. In humans developmental abnormalities attributed to the neural crest include defects of the heart (mostly of the outflow tract), craniofacial development (cleft lip/palate), peripheral nervous system (familial dysautonomia) and certain cancers including melanoma and neuroblastoma [13], [14].

During embryonic development, neural crest induction is initiated by a few signaling molecules such as BMP, FGF, and Wnt [15]–[17], which lead to the sequential up-regulation of transcription factors in neural crest precursors. The first cohort of transcription factors, including Pax3, Pax7, Zic1, Msx1 and Msx2, specify the neural plate border. This in turn initiates the expression of a second marker set, including Sox9, Sox10, FoxD3, AP-2, c-Myc, that specify the neural crest [18], [19]. These signals are further combined with additional downstream effectors to enable the proliferation, survival, detachment, migration and differentiation of neural crest cells. Even though mutations of several of these genes have been shown to alter specific neural crest derivatives, it is not yet clear what are the specific contributions of each transcription factor, nor their collaborative effects, downstream targets, or how these contribute to the production of neural crest-derived cells.

Our laboratory has identified the paired box domain transcription factor, Pax7, as one of the earliest markers of avian neural crest cells, and demonstrated its requirement for avian neural crest development [20]. However the lineage contributions of Pax7 precursors to mammalian neural crest development are unknown. In the E8 to E14 mouse embryo, Pax7 expression is detected in regions of the developing neural crest (the dorsal neural tube, cranial neural folds) [21]–[23]; regions not typical of the developing neural crest (brain, olfactory epithelium, frontonasal region, and somites) [22], [24], [25]; and in varied but restricted regions postnatally (frontonasal region, skeletal muscle satellite cells, brain and pituitary) [21], [26]–[28].

To date, transgenic mice have shown that the Pax7 lineage includes fat, dermis, muscle [29], [30]; olfactory neurons, ensheathing glia and supporting cells [25]. However, the Pax7 expression profile suggests a broader lineage potential for Pax7 descendants, like that seen for its closest Pax family member, Pax3 [31], [32]. Using transgenic mice, we expand the repertoire of lineages contributed by Pax7, with emphasis on the neural crest. In doing so, we identify some interesting similarities between the Pax3 and Pax7 lineages, within and outside of the typical neural crest.

**Figure 1:**
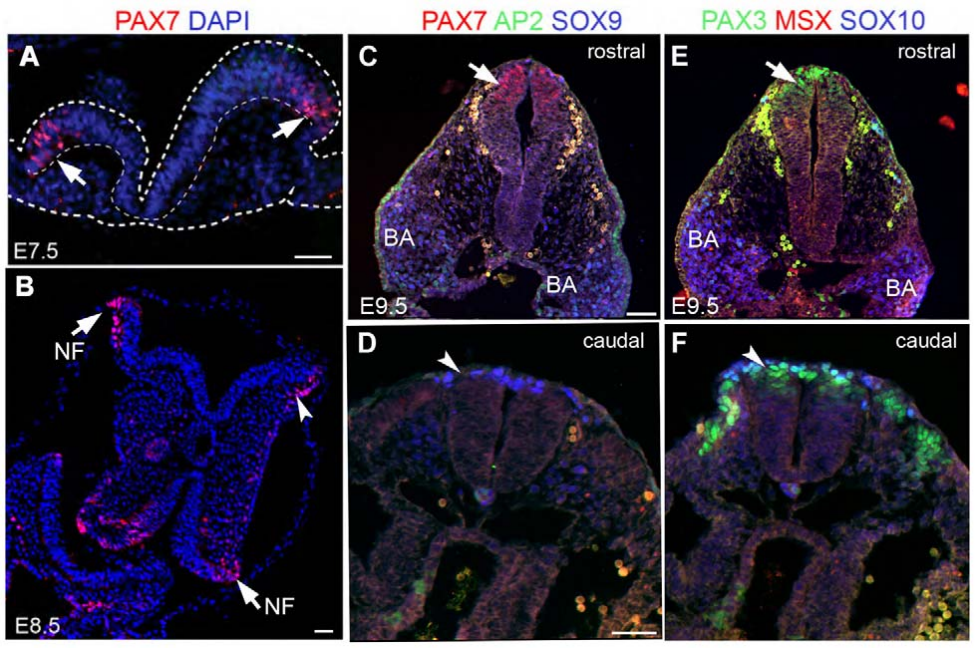
Spatiotemporal expression of neural crest markers including Pax7 in E7.5 to E9.5 mouse embryos. In C57BL/6 embryos, immunohistochemistry detects Pax7 (red) in A,B) the E7.5 to E8.5 lateral neural folds (NF; arrows); B) the E8.5 cephalic mesenchyme (arrowhead). Dapi (blue) is a nuclear stain. C,E) and D,F) are serial sections from the same axial level at E9.5. C) Pax7 is detected in the rostral (arrow), but not D) caudal (arrowhead), dorsal neural tube, whereas F) Pax3 (green) is more highly expressed in the caudal (arrowhead) and not E) rostral (arrow) neural tube. C,D) AP2-α+ cells (green) are seen mostly in the ectoderm; rare AP2-α+ cells are seen together with Sox9+ (blue) and E,F) Sox10+ cells (blue) in the branchial arches (BA); Msx1+ cells (red) were undetected in these sections. Note that rounded cells adjacent to the neural tube are background, which appear also in the negative controls. Scale bars in A–D −100 um.

## Results

### Spatiotemporal Expression of Neural Crest Markers Including Pax7 in the Early Mouse Embryo

Pax7 is one of the earliest markers of, and required for, neural crest development in the chick [20], [33]. However, the contribution of Pax7 precursors to mammalian neural crest tissues has not been determined. In the mouse, *in situ* showed Pax7 expression beginning at E8.0 through E14, in the closed neural tube, developing brain, nasal region and somites [21]–[23]. Using immunohistochemistry on E7.5 to E9.5 C57BL/6 embryos, we determined the spatiotemporal expression of Pax7 protein at the cellular level. We first detected Pax7 protein prior to neural tube closure at E7.5, at the tips of the lateral neural folds (Fig. 1A). At this early stage, Pax7+ cells form only a subpopulation of the larger expression domain of the neural crest, as indicated by the neural crest markers, AP2-α, Sox10 and Pax3 (Casey DelConte and Martín García-Castro, unpublished data). By E8.5 both the neural folds and cephalic mesenchyme contained small numbers of Pax7+ cells (Fig. 1B). Pax7 expression was more widely detected at E9.5 in the rostral neural tube (Fig. 1C), but markedly absent from the caudal neural tube (Fig. 1D), consistent with the suggestion that the Pax7 lineage contributes more highly to cranial regions [23]. In contrast to Pax7 expression, Pax3 expression was more readily detected in the caudal compared to rostral neural tube (Fig. 1E,F). Sox9+ (Fig. 1C,D) and Sox10+ cells (Fig. 1E,F) were seen mostly in the branchial arches (together with AP2-α+ cells), with rare cells in the dorsal neural tube. AP2-α+ cells were also detected in the developing ectoderm (Fig. 1C,D). From E10 to postnatally, Pax7 expression continues in the frontonasal region [21], [24], [25], skeletal muscle and in the pituitary gland [21], [26]–[28], confirming earlier studies (data not shown). These results indicate the early expression of Pax7, in more rostral regions of the mouse, which includes a subpopulation of presumptive neural crest precursors.

**Figure 2:**
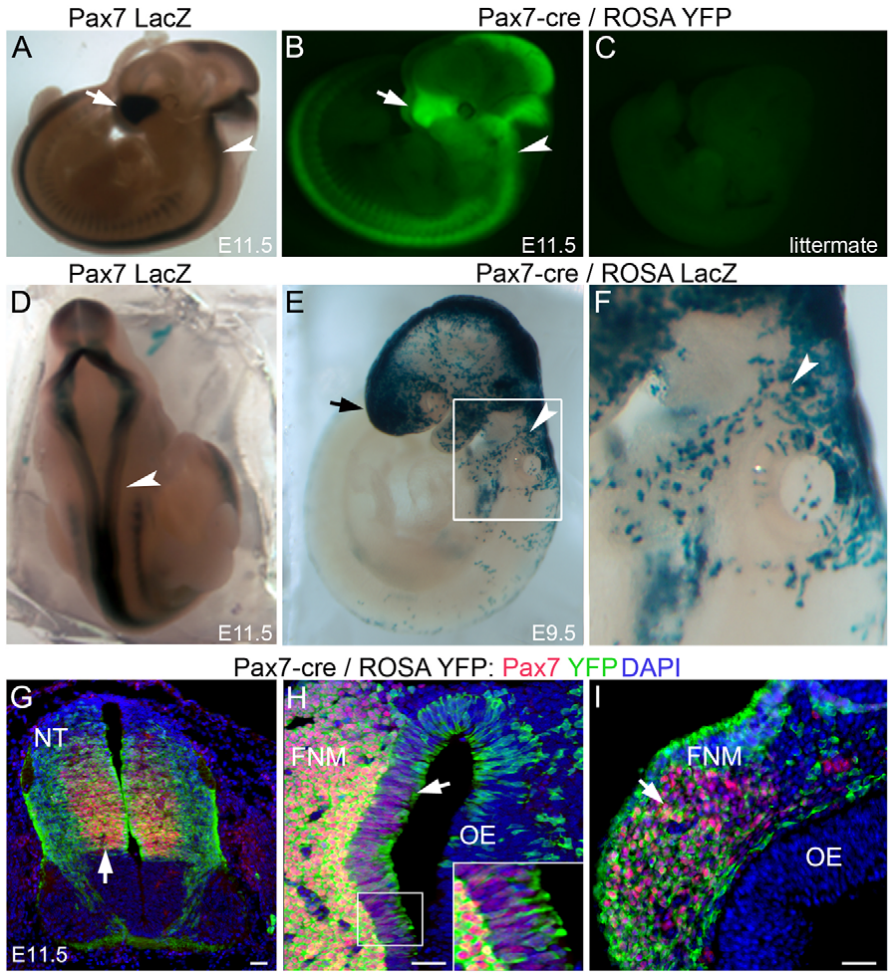
Pax7-cre/reporter mice for lineage tracing neural crest derivatives. A,B,D) Equivalent Pax7 expression domains are seen in the frontonasal region (arrows) and neural tube (arrowheads) of E11.5 mouse embryos from different Pax7 transgenic lines: A,D) Pax7 LacZ (LacZ stain) and B) Pax7-cre/ROSA YFP (endogenous YFP expression). C) A littermate control, lacking YFP expression. E,F) E9.5 Pax7-cre/ROSA LacZ embryos show LacZ stain in neural crest regions, such as the frontonasal region (arrow) and cells leading to the pharyngeal arches (arrowheads). Box in (E) magnified in (F). G–I) In E11.5 Pax7-cre/ROSA YFP embryos, co-expression of Pax7 (red) and YFP reporter (green) can be detected in G) the neural tube (NT), H,I) olfactory epithelium (OE) and frontonasal mesenchyme (FNM; arrows). Inset highlights boxed region. Scale bars in G–I 100 um.

### Pax7-cre/reporter Mice for Lineage Tracing Neural Crest Derivatives

To determine if Pax7 could identify precursors whose progeny contribute to neural crest tissues, we crossed Pax7-cre transgenic mice, driving Cre recombinase from the Pax7 locus, to *Gt(ROSA)26Sor^tm(EYFP)Cos^* reporter mice (hereafter termed ROSA YFP) [34], to fate map Pax7 descendants. Following Cre-mediated recombination in double transgenic Pax7-cre/ROSA YFP mice, a floxed stop cassette is excised allowing for the continued expression of YFP in Pax7-expressing precursors and their progeny, even after Pax7 expression ceases. The transgenic reporter line *Gtrosa26^tm1Sor^* (hereafter termed ROSA LacZ), that expresses ß-galactosidase protein from the ROSA26 locus [35], produced a similar expression pattern after Pax7-cre mediated excision.

**Figure 3:**
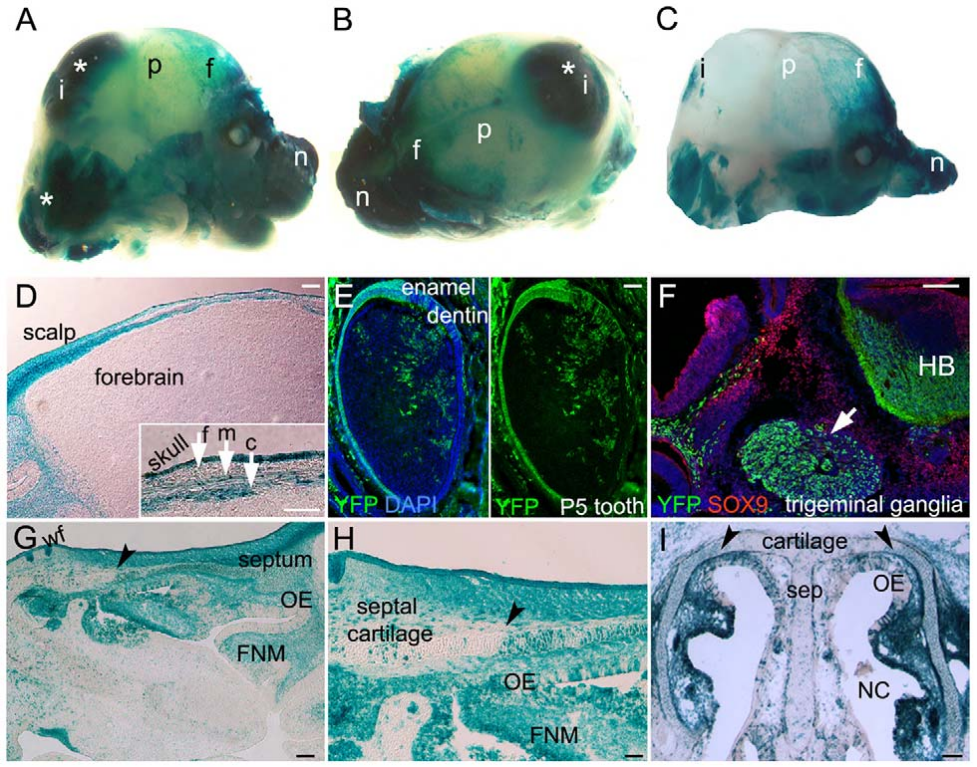
The Pax7 lineage contributions in the cranial region. LacZ/YFP expression in E15.5 Pax7-cre/ROSA reporters is detected in multiple cranial regions that include: A,B) the brain (asterisks), skull, skin and nose; A–C) in the nasal (n) region, interparietal (i), parietal (p) and frontal (f) bones of the skull (viewed in (C) with brain removed); D) frontal bone (f), meninges (m) and connective tissue (c; arrows inset). E,F) YFP reporter (green) is detected in teeth (postnatal day 5 (P5)), and the trigeminal ganglia (arrow, F) near Sox9+ cells (red, F). DAPI nuclear stain in blue. G–I) Pax7-cre/ROSA LacZ mice express LacZ in the nasal cartilage, cells of the frontonasal mesenchyme (FNM), olfactory epithelium (OE) and whisker follicles (wf). Arrowheads in (G–I) show distinct border between dense and diffuse recombination+ cells in cartilage. Note (C) has been cropped in Photoshop for aesthetics. Sections are sagittal (D–H) and coronal (I). HB –hindbrain, NC –nasal cavity, sep -septum. Scale bars in D,F,G,I-500 um; E,H, inset -200 um.

We determined if Pax7-cre/ROSA YFP mice faithfully reported according to endogenous Pax7 expression, by comparing the YFP expression pattern, to that seen in multiple independent transgenic lines, including some whose reporter expression occurred independent of recombination. Similar expression patterns were seen in age-matched Pax7 LacZ (Fig. 2A,D), Pax7-cre/ROSA YFP (Fig. 2B), Pax7-cre/ROSA LacZ (data not shown and [36]) and Pax7-ZsGreen [37] transgenic mice, each recapitulating the expression domains of endogenous Pax7 [23], [38]. Additionally, in E11.5 Pax7-cre/ROSA YFP embryos, co-expression of Pax7 protein with YFP was detected in the dermomyotome (not shown), olfactory epithelium, neural tube, and frontonasal mesenchyme (Fig. 2G–I) [25]. Littermate controls of Pax7-cre/ROSA YFP embryos, having only a single or entirely lacking transgenic alleles, did not express YFP (Fig. 2C).

**Figure 4 pone-0041089-g004:**
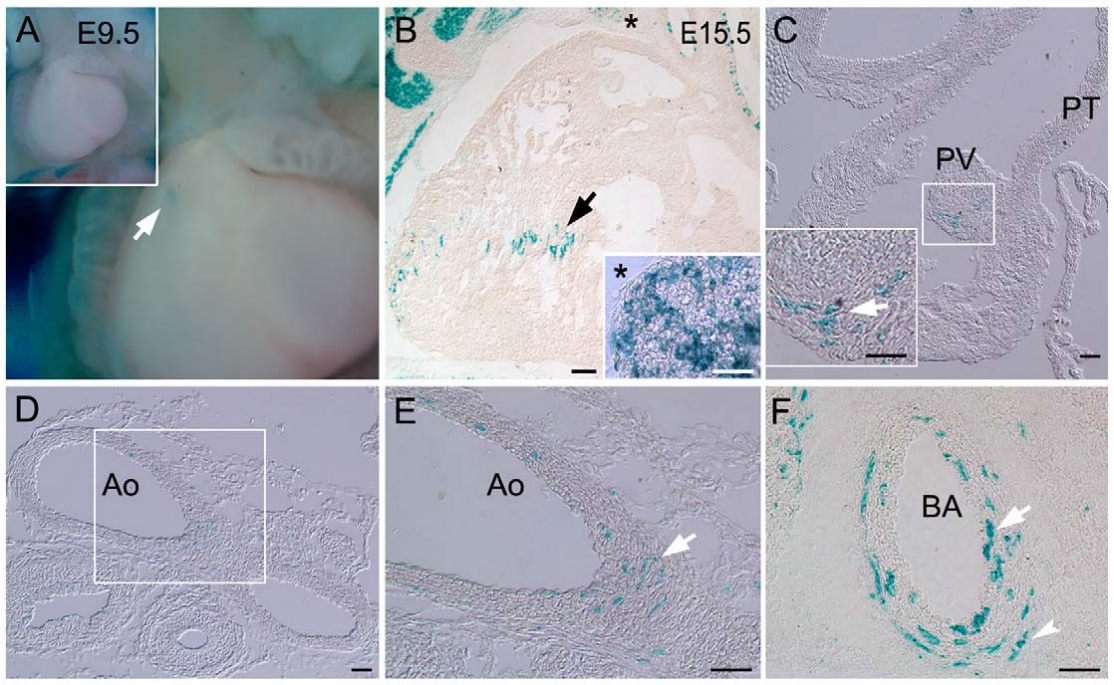
Limited cardiovascular tissue derived through the Pax7 lineage. A–F) Recombination+ cells (LacZ+) are found in the heart and blood vessels of Pax7-cre/ROSA LacZ mice. A) Wholemount E9.5 embryos show faint LacZ+ cells in the heart (arrow). Inset shows lower magnification of panel. B–F) E15.5 sections show small populations of LacZ+ cells (arrows) in the B) heart, thymus (asterisk and inset), C) pulmonary valve (PV), D,E) aorta (Ao) and F) inner (arrow) through outer (arrowhead) layers of the brachiocephalic artery (BA). Boxes show magnified region in separate panel. PT –pulmonary trunk. Scale bars in B −500 um; insets, C–F −200 um.

Consistent with Pax7 expression, at E8.5 in Pax7-cre/reporter embryos, a few YFP+ cells were first detected in the cephalic mesenchyme and lateral neural folds [25] (and data not shown). By E9.5, Pax7 descendants (LacZ+) were detected in the midbrain, hindbrain, dorsal neural tube, frontonasal region, and in migratory neural crest cells emigrating from the dorsal neural tube to the branchial arches (Fig. 2E,F). Combined our results provide evidence validating the use of Pax7-cre/reporter mice to follow Pax7 descendants in the neural crest. Using these Pax7-cre/reporter mice we traced, the cranial, cardiac and trunk neural crest lineages.

**Figure 5 pone-0041089-g005:**
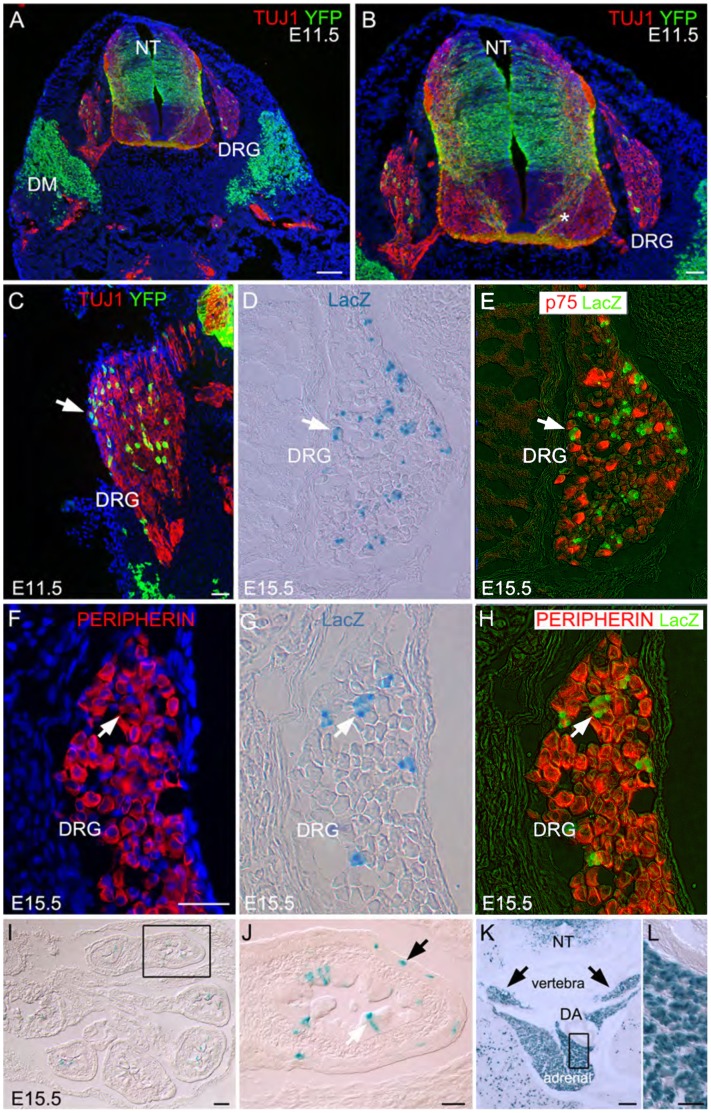
Pax7 contributions to trunk neural crest. A–C) At E11.5 and D–H) E15.5 in Pax7-cre/ROSA reporter mice, Pax7 descendants (YFP+ or LacZ+, respectively) are detected in the neural tube (NT), including commissural axons (asterisk), dermomyotome (DM) and dorsal root ganglia (DRG). A–C) Only a subset of the total DRG neurons (TUJ1+, red) arise through the Pax7 lineage (YFP+, arrow). Neurons throughout the DRGs express E) P75 (red) and F,H) peripherin (red), including D–H) LacZ+ Pax7 descendants (arrows). In E,H), the LacZ signal (green) was pseudocolored in Photoshop. I,J) Within the physiological umbilical hernia, subsets of Pax7 descendants (LacZ+) are detected in the lining of the midgut (white arrow) and presumptive enteric neurons (black arrow, J), K,L) sympathetic tissue (arrows) and adrenal gland (all E15.5). Boxes show magnified region in adjacent panel. DA-dorsal aorta. Scale bars in A,I,K -500 um; B,C,F,J,K magnified region −200 um.

### The Pax7 Lineage Contributions in the Cranial Region

In the cranial region of E15.5 Pax7-cre/reporter mice, both neural crest and non-neural crest recombinants were detected (Fig. 3). LacZ+ cells were seen in the brain, skin and nose (Fig. 3A,B). In the skull, cranial crest derivatives arising via the Pax7 lineage included subpopulations of cells of the frontal, parietal and interparietal bones (Fig. 3A–D). Below the skull, recombinants were detected in the connective tissue and meninges (Fig. 3D). In Pax7-cre/ROSA YFP mice, YFP+ cells were seen in postnatal day 5 teeth (Fig. 3E), and at E15.5, in the trigeminal ganglia and hindbrain, independent of Sox9+ precursors (Fig. 3F). Sagittal sections of the head showed LacZ+ cells in the presumptive neural crest tissues of the whisker follicles, the frontonasal mesenchyme, olfactory epithelium and cartilage of the septum (Fig. 3G–I). Interestingly, recombinant cells were more highly detected in the lateral cartilage (93.5±0.8%, p<0.001) compared to medial cartilage of the septum (14.9±1.3%) (Fig. 3G–I). These results show the widespread contributions of the Pax7 lineage to numerous cranial tissues, some with distinct spatial patterns.

**Figure 6:**
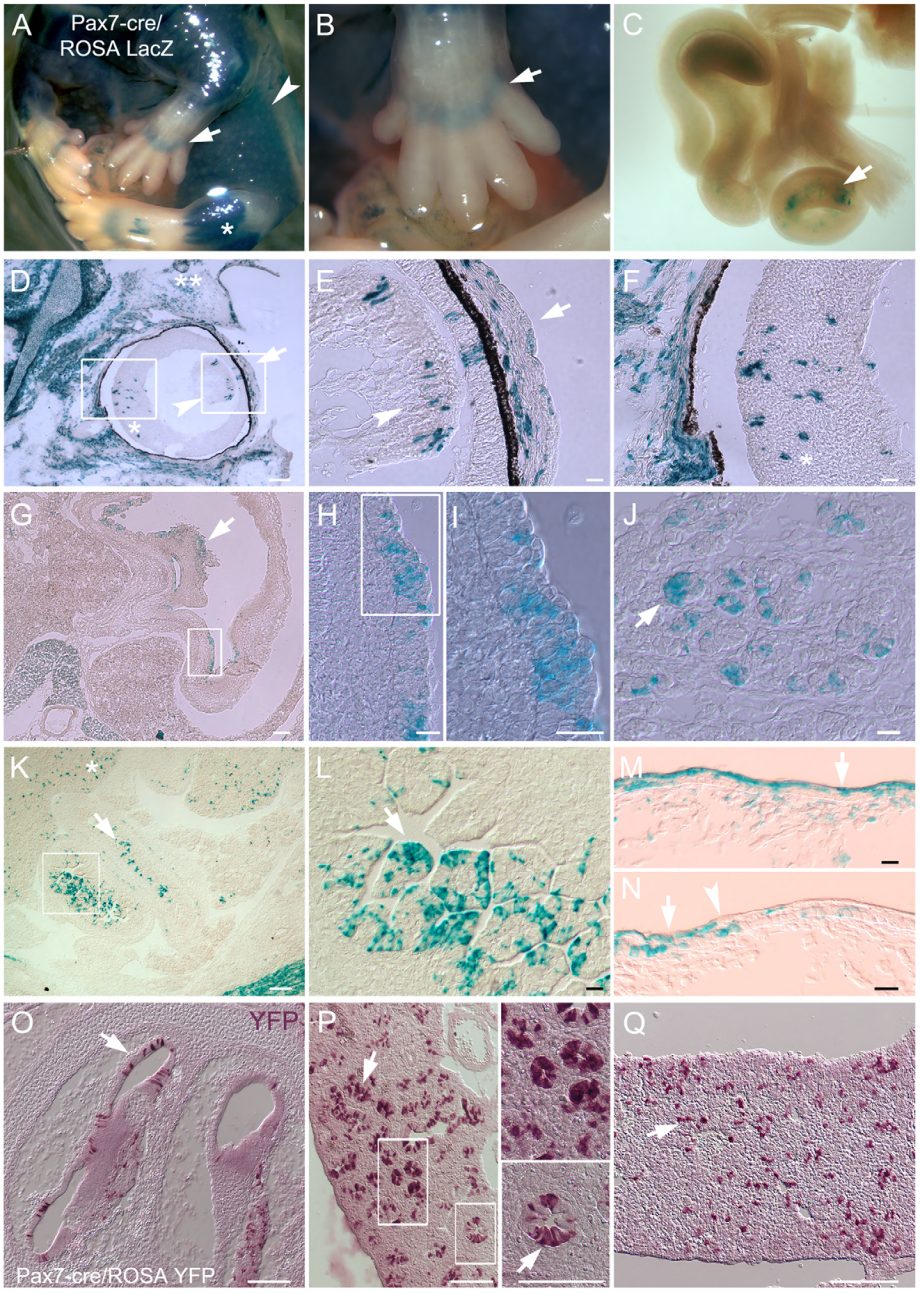
The Pax7 lineage outside of the neural crest. In E15.5 Pax7-cre/reporter embryos, LacZ+ Pax7 descendants are seen in: A) the skin (arrowhead) and skeletal muscle (asterisk); A,B) restricted regions of the carpal bone primordium (arrows) and C) portions of the wholemount gut (arrow). In sections, a subset of LacZ+ cells are detected in: D–F) the eye; the presumptive eyelid muscle (double asterisk), in the lens (arrowhead) and its surrounding tissue (arrow), and neural retina (asterisk); G–I) the lining of the stomach (arrow); J) the pancreas (arrow identifies presumptive acinar cells); K,L) the lining of the gut (arrow; asterisk denotes the liver); and M,N) the skin (arrow), where a distinct border distinguishes the Pax7 lineage from non-lineage Pax7 (arrowhead). O–Q) YFP+ cells visualized with non-fluorescent VIP are detected in: O) the cochlea of the ear; P) lining of lung; and Q) liver (arrows for all). Both YFP and LacZ Pax7-cre/reporters gave the same results, as did fluorescent and non-fluorescent detection methods. Boxes show magnified region in adjacent panel. Scale bars in D,G,K,O–Q are 500 um; E,F,H–J, L–N are 100 um.

### Limited Cardiovascular Tissue Derived through the Pax7 Lineage

Compared to the cranial neural crest, and in contrast to the Pax3 lineage [31], there were limited contributions of the Pax7 lineage to cardiovascular tissues. Faint LacZ+ cells were seen in wholemount hearts from E9.5 Pax7-cre/ROSA LacZ embryos (Fig. 4A). Although in sections of E15.5 samples, few LacZ+ cells were readily detected in the heart and thymus (Fig. 4B), the pulmonary valve (Fig. 4C), the arch of the aorta just proximal to the entrance site for the ductus arteriosus (Fig. 4D,E), and in the brachiocephalic (Fig. 4F) and carotid arteries (data not shown). These results indicate a limited contribution of the Pax7 lineage to cardiovascular tissues and the cardiac neural crest.

### Pax7 Contributions to Trunk Neural Crest

Trunk sections of Pax7cre/ROSA YFP and LacZ embryos showed recombinant+ cells (YFP+ or LacZ+) in the dermomyotome, neural tube, commissural axons, and dorsal root ganglia (DRGs; Fig. 5A–H). At E11.5 (Fig. 5A–C) and E15.5 (Fig. 5D–H), the neuronal antigens TUJ1 (Fig. 5A–C), P75 (Fig. 5E) and peripherin (Fig. 5F,H) were widely expressed throughout DRG neurons. However, only a subset of DRG neurons (4.5±0.7%; 32/702 TUJ1+ neurons counted), arises via the Pax7 lineage (Fig. 5A–H). Pax7 progeny also included subsets of presumptive enteric neurons surrounding the outer portion of the gut, and epithelial cells lining the gut (Fig. 5I,J), sympathetic tissue and cells of the adrenal gland (Fig. 5K,L). These results indicate a broad, albeit restricted, contribution to the trunk neural crest by the Pax7 lineage.

### The Pax7 Lineage in Non-neural Crest Regions

Our focus was primarily on neural crest derivatives, however we examined tissues not commonly associated with the neural crest, as some were previously reported in the Pax3 lineage [31], the closest Pax family member to Pax7. After histochemical staining, E15.5 Pax7-cre/ROSA LacZ embryos appeared mostly LacZ+ (Fig. 6A). In wholemount, Pax7 descendants were detected in the lung, liver, thymus (data not shown), skeletal muscle (Fig. 6A) and gut (Fig. 6C). Unlike the Pax3 lineage, Pax7 descendants were not detected in the kidney. In the paws, LacZ+ cells were restricted to the presumptive carpal bone primordium (Fig. 6A,B).

Sections revealed a more detailed analysis of the cells and tissues to which Pax7 descendants contribute. A striking finding for the Pax7 lineage was their contribution to only a subset of seemingly homogeneous cell populations within certain tissues. In the eye LacZ+ Pax7 descendants contributed to subsets of cells in and around the lens, neural retina, presumptive ocular muscles and eyelids (Fig. 6D–F). A similar trend was found in the stomach lining (Fig. 6G–I), pancreas (including acinar cells, Fig. 6J), gut lining (Fig. 6K,L) and skin (Fig. 6M,N), where each contained a population of cells formed in part by Pax7 derivatives. In addition to the underlying connective tissue, the skin contained Pax7 derivatives in the epidermis and dermis that showed a distinct regional boundary, with positive cells in the back skin that went midway around to the belly (Fig. 6M,N). Non-fluorescent detection of YFP revealed Pax7 lineage contributions to lung bronchioles (Fig. 6P), liver (Fig. 6Q) and, consistent with the detection of lineage+ cells in the E9.5 otic vesicle (Fig. 2E,F), cells in the cochlea of the ear (Fig. 6O). Similarly, Wnt-1-cre and Pax3-cre lineages contribute to the otic vesicle and inner ear [32]. These results show a previously unappreciated diversity for the Pax7 lineage throughout the body, including overlap with some of the Pax3 and Wnt-1 lineages [31].

## Discussion

Using transgenic mice, we mapped the Pax7 lineage focusing on the neural crest. Consistent with the predominance of craniofacial defects seen in Pax7 mutants [23], we found a higher contribution of Pax7 descendants in the cranial compared to either cardiac or trunk neural crest (Fig. 3–5). More globally, Pax7 descendants showed two interesting traits: 1. a regional pattern especially evident in the head cartilage (Fig. 3), and 2. within specific tissues, contributions to only a subset of cells in a presumed homogeneous population (Fig. 3–6). Our results reveal a previously unappreciated widespread distribution of Pax7 descendants, within and beyond the neural crest.

Although neural crest derivatives were prevalent in the Pax7 lineage, many Pax7 descendants were found outside of the ectoderm-derived neural crest tissues. The lining of the stomach, pancreas, gut and lungs each contained Pax7 descendants, as did cells in the liver (Fig. 6), indicating endoderm derivatives. Thus, together with the mesoderm derivatives, muscle and fat [29], [30], Pax7 precursors contribute to tissues derived from all three germ layers.

### Pax7 in the Neural Crest Lineage: Comparisons to Wnt1-cre Mice

Previous studies have fate mapped the neural crest lineage using transgenic mice [39], [40], with the most common and extensive neural crest labeling detected with the Wnt1-cre driver [41]–[43]. Neural crest cells targeted by Wnt1-cre label descendants in craniofacial structures, olfactory, cardiac, endocrine, peripheral and enteric nervous system derivatives [41]–[44]. Here, in neural crest derivatives, we highlight the most pronounced distinctions between Wnt1 and Pax7 descendants.

Like Wnt1-cre, Pax7-cre mice targeted neural crest cells at all axial levels, but in contrast labeled only relatively small subpopulations of certain neural crest derivatives (Fig. 3–5). Wnt1-cre mice label more cells in the frontal and interparietal bones of the skull [42] main arteries surrounding the heart [43], and in the DRGs [45]. Pax7, but not Wnt1 descendants, contribute to the parietal bone in a mosaic pattern, although these are likely derived from the mesoderm [42], [46]. Such a mosaic pattern is evident in multiple tissues (Fig. 5,6; olfactory and gut epithelia, inner ear, lung, pancreas, etc.), and consistent with patterns seen using several different Cre drivers (Pax7-, Wnt-1-, Pax3-, Bmi-, Id2- cre) [25]. These patterns suggest the input of multiple progenitors to certain tissues, thus allowing for compensation by alternate progenitors after progenitor loss, to maintain tissue integrity. This is supported by the only mild defects seen in Pax7 mutants at birth [23].

Despite these differences, in certain cranial crest derivatives, like the frontonasal mesenchyme and olfactory ensheathing glia, similar proportions of labeled cells are seen in both Wnt1-cre and Pax7-cre/reporter mice [25], [44], [46], [47]. This pattern suggests a convergence of the Pax7 and Wnt1-derived neural crest lineages, at least in some regions.

### Regional Restrictions in the Pax7 Lineage

Of particular interest regarding the Pax7 neural crest lineage is the distinct regional restriction of labeled cells where approximately 6 times more Pax7 descendants populated the lateral compared to the central head cartilage (Fig. 3; 93.5% vs 14.9%). This pattern could arise either through the seeding of more Pax7 derivatives in the lateral compared to medial septum or by the preferential expansion and/or survival of the lateral Pax7 derivatives. A more detailed examination using BrdU labeling and apoptosis assays would provide a better understanding of the development of this head cartilage.

### The Pax7 Compared to Pax3 Lineage

Of the 9 mammalian Pax genes, Pax7 is most structurally similar to Pax3, each having a paired domain, octapeptide region and homeodomain, and together form the Pax subgroup III [48], [49]. Since Pax7 and Pax3 have only partially overlapping expression domains, one would predict both common and distinct descendants. When *Cre* is inserted into exon 1 of Pax3, and thus faithfully recapitulates the endogenous Pax3 expression domain, Pax3 descendants can be found in the dorsal neural tube, somites, skeletal muscle, adrenal gland and facial structures [31]. Here we report a very similar pattern of derivatives generated by Pax7 progenitors (Figs 2, 3, 4, and 5). Additional derivatives common to both Pax7 and Pax3 lineages include the ear, colon epithelia, stomach (Fig. 5,6) and olfactory epithelium [31], [50], [51]. Unlike Pax3 descendants [31], Pax7 descendants were not detected in the kidney.

In certain tissues, like the enteric plexus and cardiac neural crest, the Pax7 lineage was less prevalent than the Pax3 lineage [31]. This is reflective of the loss of enteric ganglia and severe cardiovascular defects seen in homozygous Pax3 *“Splotch”* mutants [51]–[53], but absent from Pax7 mutants [23].

The Pax7 lineage was detected in some tissues where Pax3 descendants have not been reported - the presumptive carpal cartilage, lens and neuroepithelium of the eye, cells in the liver, pancreas, skin epithelium, and lung bronchioles (Fig. 6, and data not shown). The differences between the Pax7 and Pax3 derivatives reflect the divergent regulation of Pax7 compared to Pax3 expression. Given this diverse range of Pax7 derivatives it is intriguing to speculate that defects in survival and/or turnover of cells in the pituitary [26], lung, liver, gut, etc., may similarly, and in addition to muscle stem cell defects, contribute to the stunted neonatal size and death of Pax7 mutants.

### Pax7 Progenitors -multipotent or Restricted?

Pax7 progenitors contribute to many different tissues and cell types, representing derivatives that are distributed throughout the body from each of the three germ layers. Are Pax7 progenitors then multipotent, or do they instead represent varying yet more restricted progenitors? The Cre-LoxP lineage-tracing approach used in this study loyally marks progenitors that expressed Pax7, however it does not allow one to match the precise time and region of emergence of a Pax7+ precursor with its contributions to specific tissues and cell types. Timely inducible promoters and/or enhancer specific strategies offer better-fitted alternatives for this purpose. A previous study induced Pax7-cre expression with tamoxifen at varying developmental stages, and tested for reporter expression one day later [30]. Injections on E7.5 (the first time point to reveal Pax7 derivatives), showed a few labeled cells near the dorsal neural tube, indicative of neural crest, whereas cells labeled on E8.5 included the cephalic neural crest and craniofacial region (in addition to parts of the brain, spinal cord, and somites). After injections between E9.5 to E13.5, labeling persisted in the craniofacial region, spinal cord and somitic tissues. Our detection of Pax7 protein at E7.5 to E9.5 in the neural folds, cephalic mesenchyme and rostral dorsal neural tube (Fig. 1), and later in the frontonasal mesenchyme and olfactory epithelium (Fig. 2 and [25]), together with our lineage tracing results, are in agreement with and compliment this study. In most of the tissues with Pax7 lineage contributions, we did not detect Pax7 expression and hence could not identify a tissue-specific Pax7 precursor. These findings are also supported by the induction experiments [30] and demonstrate the dynamic nature of Pax7 progenitors during different stages of development. That these labeled cells are indeed Pax7 descendants is likely, since our labeling pattern is consistent with multiple transgenic lines, even those whose reporter expression is independent of recombination (Fig. 2) [23], [36]–[38]. Additionally, YFP could be detected coincident with endogenous Pax7 expression (Fig. 2 and data not shown) [25]. Future studies using clonal analyses of individual Pax7+ progenitors will reveal their potency and help to dissect the molecular pathways leading to the differentiation of specific cell types.

### Conclusion

Here we used Pax7-cre/reporter double transgenic mice to identify the Pax7 lineage in the neural crest. We found the Pax7 lineage contributed more highly to the cranial compared to either cardiac or trunk neural crest. Two patterns formed by Pax7 descendants were notable: 1. a regionally restricted pattern in the cranial cartilage and 2. contributions to only a subset of seemingly identical cells, within a given tissue. Outside of the neural crest, the Pax7 lineage was widespread, contributing to sensory organs, the lining of organs, and parts of the digestive system. We thus uncover a previously unappreciated broad tissue and cell diversity for the Pax7 lineage, which may help to better understand the development and function of these structures. Our results additionally, have implications for regenerative medicine, as future studies dissect the molecular regulation required to direct the differentiation of Pax7 precursors to specific cell types.

## Materials and Methods

### Ethics Statement

All experimental procedures were specifically approved (protocol # 2009-11115) and performed in accordance with the Yale Animal Resources Center and Institutional Animal Care and Use Committee policies.

### Transgenic Mice

*Pax7 ^ICNm^* mice (C57Bl/6; termed *Pax7-cre* throughout the paper), obtained from Mario Capecchi [36], were crossed with either C57Bl/6 female *Gt(ROSA)26Sor^tm(EYFP)^* ^Cos^ mice [34], expressing enhanced yellow fluorescent protein from the ROSA26 locus (termed ROSA YFP throughout the paper) or *Gtrosa26^tm1Sor^* [35], expressing ß-galactosidase protein from the ROSA26 locus (termed ROSA LacZ throughout the paper). Mouse reporter lines were gifts from Diane Krause. Both reporter lines produced a similar reporter expression pattern after Pax7-cre mediated excision. Both *Pax7-cre/ROSA YFP* and *Pax7-cre/ROSA LacZ* double transgenic mice, where Cre recombinase expression is under the control of the endogenous Pax7 regulatory elements, express the reporters without disrupting Pax7 function [36]. Mice were genotyped using PCR for Pax7, the Cre knock-in allele of Pax7 [36], and the modified ROSA26 allele containing either YFP [34], or LacZ [35]. Pax7 primers located within Pax7 exon 10 5′-GCTCTGGATACACCTGAGTCT-3′; 5′-TCGGCCTTCTTCTAGGTTCTGCTC-3′ (ck118 and ck256 respectively; wildtype 465 bp product) were combined with an *IRES-Cre* primer 5′-GGATAGTGAAACAGGGGCAA-3′ (ck172; 340 bp product) [36]; ROSA YFP primers: Rosa1 5′-AAAGTCGCTCTGAGTTGTTAT-3′ and Rosa3 5′-GGAGCGGGAGAAATGGATATG-3′ (wildtype ROSA locus, 650 bp product); Rosa3 and YRF 5′-CGACCACTACCAGCAGAACA-3′ (ROSA26 YFP, 850 bp product); ROSA LacZ primers: Rosa1 5′-AAAGTCGCTCTGAGTTGTTAT-3′ and Rosa2 5′-GCGAAGAGTTTGTCCTCAACC-3′ (wildtype ROSA locus 650 bp, ROSA26 LacZ 350 bp product). PCR parameters: 35 cycles of 95°C 30 seconds, 58°C 30 seconds, 72°C 60 seconds. Efficiency of Cre-lox recombination was verified by the specific co-localization of Pax7 with YFP proteins in *Pax7-cre/ROSA YFP* embryos and the similar reporter expression patterns detected in Pax7-LacZ [38], Pax7-Zs-green [37] and Pax7-cre/*Gtrosa26^tm1Sor^* mice [36]. For each time point, at least three Pax7-cre/reporter positive embryos or mice were analyzed.

### Tissue Preparation

Mice were sacrificed in a CO_2_ chamber, perfused with cold PBS and 4% paraformaldehyde (PFA) in PBS and post-fixed in 4% PFA at 4°C [54]. Embryos were immersion-fixed in 4% PFA overnight. The day of vaginal plug was defined as E0.5. Tissues were cryoprotected in sucrose, embedded in either Tissue-Tek medium (OCT; Sakura Finetek, Torrance, CA) or gelatin and frozen in liquid nitrogen. 12 µm sections were stored at –20°C for subsequent analysis.

### Immunohistochemistry

Sections were immersed in PBS, permeabilized in 0.1% Triton-X-100/PBS and blocked with 4% normal serum prior to primary antibody incubation. Secondary antibodies (1∶200) used were of specific isotypes conjugated to biotin (Vector labs), Alexa 568 or Alexa 488 (Invitrogen Molecular Probes). Primary antibodies used: mouse anti-rat βIII tubulin (neuron-specific tubulin-TUJ1, 1∶500) Covance; mouse anti -Pax7, Developmental Studies Hybridoma Bank (developed by Kawakami, A), rabbit anti-GFP (1∶400, to detect YFP), Millipore. Note: all YFP panels show anti-GFP immunofluorescence that was confirmed using non-fluorescent VIP immunohistochemistry with anti-GFP antibodies (as seen in Fig. 6O–Q), with the exception of the YFP-containing panels in Fig. 2B,C which detect endogenous YFP fluorescence. Nuclei were stained with 0.5 ug/ml diaminopyridine imidazole (DAPI) and sections coverslipped in Vectashield (Vector Laboratories, Burlingame, CA) for fluorescent antigens or 50% glycerol for VIP. All images were visualized with either a Nikon dissecting scope or Nikon Eclipse 80 i microscope using a SPOT camera (SPOT Diagnostic Instruments Inc.) with SPOT software (v4.5) and compiled using Adobe Photoshop CS4.

### Histochemistry

Lac Z histochemistry was performed on wholemounts or cryosections postfixed in 4% PFA, permeabilized in 0.1% Triton-X-100 and washed in PBS before adding staining buffer (2 mM MgCl_2_, 0.01% deoxycholate, 0.02% Nonidet-P40, and 100 mM NaPO_4_, pH 7.3) containing 1 mg/mL X-gal, 5 mM potassium ferrocyanide, and 5 mM potassium ferricyanide. Staining proceeded either on ice for wholemounts or at 37°C for sections, protected from light for 1–10 hours. Negative controls did not demonstrate β-galactosidase staining and included C57BL/6 non-transgenic mice and Pax7-cre transgenic mice without the ROSA reporter transgene. Results were confirmed using antibodies to β-galactosidase.
